# The Impact of Linguistic Signals on Cognitive Change in Support Seekers in Online Mental Health Communities: Text Analysis and Empirical Study

**DOI:** 10.2196/60292

**Published:** 2025-01-14

**Authors:** Min Li, Dongxiao Gu, Rui Li, Yadi Gu, Hu Liu, Kaixiang Su, Xiaoyu Wang, Gongrang Zhang

**Affiliations:** 1 School of Management Hefei University of Technology Hefei China; 2 Center for Mental Health Education University of Shanghai for Science and Technology Shanghai China; 3 School of Management Southeast University Nanjing China; 4 The 1st Affiliated Hospital Anhui University of Traditional Chinese Medicine Hefei China

**Keywords:** mental health, online communities, cognitive change, signaling theory, text analysis

## Abstract

**Background:**

In online mental health communities, the interactions among members can significantly reduce their psychological distress and enhance their mental well-being. The overall quality of support from others varies due to differences in people’s capacities to help others. This results in some support seekers’ needs being met, while others remain unresolved.

**Objective:**

This study aimed to examine which characteristics of the comments posted to provide support can make support seekers feel better (ie, result in cognitive change).

**Methods:**

We used signaling theory to model the factors affecting cognitive change and used consulting strategies from the offline, face-to-face psychological counseling process to construct 6 characteristics: intimacy, emotional polarity, the use of first-person words, the use of future-tense words, specificity, and language style. Through text mining and natural language processing (NLP) technology, we identified linguistic features in online text and conducted an empirical analysis using 12,868 online mental health support reply data items from Zhihu to verify the effectiveness of those features.

**Results:**

The findings showed that support comments are more likely to alter support seekers’ cognitive processes if those comments have lower intimacy (β_intimacy_=–1.706, *P*<.001), higher positive emotional polarity (β_emotional_polarity_=.890, *P*<.001), lower specificity (β_specificity_=–.018, *P*<.001), more first-person words (β_first-person_=.120, *P*<.001), more future- and present-tense words (β_future-words_=.301, *P*<.001), and fewer function words (β_linguistic_style_=–.838, *P*<.001). The result is consistent with psychotherapists’ psychotherapeutic strategy in offline counseling scenarios.

**Conclusions:**

Our research contributes to both theory and practice by proposing a model to reveal the factors that make support seekers feel better. The findings have significance for support providers. Additionally, our study offers pointers for managing and designing online communities for mental health.

## Introduction

### Background

People with psychological distress are more likely to seek support from online social platforms [1] with the advantage of connecting with a large group of individuals [2-4]. Internet use and online communication have shown the potential to improve mental health by strengthening social network relationships [5-7]. Brief, frequent, asynchronous communication on social media can provide a high level of social support [8].

The proliferation of online mental health communities, spurred by the advent of social media, has provided individuals with a platform for mutual support [9,10]. Within these communities, individuals experiencing difficulties can seek support by posting their concerns, while those who have undergone similar challenges can offer guidance through comments [11]. Analyzing support seekers’ responses to supportive comments reveals whether they experience a sense of improvement. This improvement in support seekers is characterized as a “positive cognitive change” [12-14].

We call community members who provide support to support seekers as “support providers.” They offer customized assistance according to support seekers’ unique needs [15]. Numerous studies have demonstrated that such contact through online mental health platforms can effectively improve members’ mental health and relieve mental health issues [16,17]. However, some issues persist within these communities, such as a lack of psychological counselors, the fact that most responses come from laypeople and not professionals, the fact that some responses are of low quality, the lack of active guidance, and the possibility of social stigma, among others [18,19]. These issues may prevent support seekers from seeking support for their problems, and the social stigma and cyberbullying may reduce their willingness to seek online mental health support again. Such low-quality support has a detrimental effect on both support seekers’ awareness of their mental health problems and their community loyalty [20]. Therefore, it is valuable to examine linguistic features that make support seekers feel better, as these features can guide support providers in crafting high-quality support posts, ultimately contributing to a healthier and more sustainable online mental health community.

A few researchers have studied the effects of topic consistency, language style adaptation, responder trustworthiness, emotion, and encouraging phrases on positive mental health outcomes in online mental health communities. Specific counseling techniques have been shown to increase the likelihood of positive health outcomes [21]. Peng et al [22] used the Linguistic Inquiry and Word Count (LIWC) lexicon to examine how support received by the Reddit mental health subcommunity affects satisfaction. Saha et al [23] explored the effects of topic consistency, language style adaptation, and respondent credibility of support postings on TalkLife on effective psychosocial outcomes. The characteristics of social support in mental health communities were examined by De Choudhury and De [24] from 4 perspectives: professional vocabulary, information, instrumentality, and emotion. To compare the characteristics of high-scoring and low-scoring supporting text comments, Chikersal et al [25] proposed emotions, pronouns, encouraging phrases, mental processes and behaviors, and length as variables of the strategies of supporting text comments.

Nevertheless, this body of research has some flaws. For instance, some characteristics of comments have not been researched, such as intimacy and uniqueness. Many studies deem the characteristic of abstraction as a low-scoring characteristic of support, but the specificity of the text and the effect of validation on cognitive change have not been calculated in depth. A large amount of interactive data are needed to verify the influence relationship [26]. In addition, existing research on factors that contribute to feeling better is less theoretically orientated. The prediction ability of a feature selected based on theory is superior to that of a feature selected based on experience [27]. The features suggested (and chosen) by the theory are more explicative, and the psychological and managerial ramifications of those features are well explained.

The majority of the care offered by a support provider is supplied in the form of text comments within the context of the online mental health community, using linguistic cues to elicit replies from other members. As a result, the quality of the linguistic signals more accurately reflects the degree of psychological support. Linguistic signals used to aid support seekers can be divided into emotional signals and informational signals according to Chen et al [28]. Affective signals are used to elicit or enhance the support seeker’s feelings, empathy, and sense of similarity with the support receiver, while information signals improve the observability of signals and reduce the cost of receiving and understanding the support. In this study, we used affective signals and information signals to study factors influencing cognitive change in users in online mental health communities.

### Signaling Theory

This study applied signaling theory to model factors influencing cognitive change. Spence [29] first proposed signaling theory to describe how information is transmitted from a sender to a receiver by a variety of signals, all of which are meant to produce desired results. The issue of information asymmetry between parties in many economic and social contexts is addressed by signaling theory [30], where one party has knowledge of its quality or intent that the other party does not [31]. Given that linguistic communication is the main form of interpersonal communication and that individuals do not always have access to the same information, signaling theory is closely related to the context of the online exchange of social support and the information asymmetry between social support seekers and providers [29].

The primary components of signaling theory include the sender, the receiver, and the signal. In the context of online mental health communities, the support provider is the signaler (a holder of knowledge that can aid in and alleviate psychological issues), who offers psychological support based on private information shared by a support seeker and has the intention of sharing this knowledge with the support seeker. The support seeker, in contrast, is the receiver, who needs support and posts personal information to obtain that support. Support providers use the features of the online platform to communicate, using language and signals to facilitate communication. They communicate with support seekers in an asymmetric situation in a way that will improve the seekers’ mood, namely through cognitive change. As different language elements can express various forms of information or emotions and eventually result in various outcomes, selecting appropriate language features when replying to others is crucial to help support seekers.

### Research Model and Hypotheses

**Figure 1:**
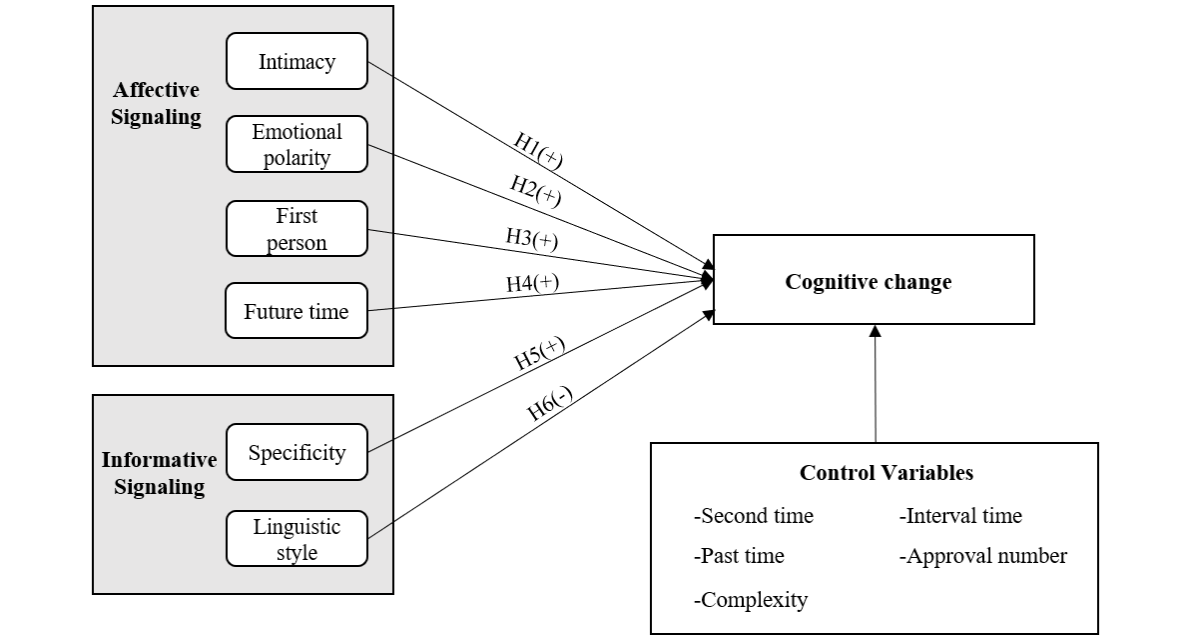
Research model. H: hypothesis.

The model of this study was developed based on signaling theory, as described earlier. Support information is the signal that the support provider sends to the support seeker, who is the signal receiver. Given existing research on the support and data characteristics of online mental health communities, as well as existing research on actual offline, face-to-face psychological counseling [32], we identified 6 characteristics: intimacy, emotional polarity, the use of first-person words, the use of future-tense words, specificity, and linguistic style. Among these characteristics, intimacy, emotional polarity, the use of first-person words, and the use of future-tense words are affective signals, while specificity and linguistic style are information signals. Figure 1 shows the complete study model.

#### Intimacy

Closeness in relationships relates to how emotionally invested and connected a person is with other participants [33,34]. It also indicates social interaction linkages. A person connects with others through interdependence, passion, and sharing [33,35,36]. By influencing social support, intimate relationships can improve mental and physical health and have a positive impact on well-being [37]. The expression of an intimate relationship can improve a person’s happiness and social interactions [38]. Intimacy has also been shown to be associated with building social capital and improving mental health [39]. A strong intimate relationship has a higher chance of receiving social support than one with poor ties [40]. Online mental health platforms provide support seekers the channel to read empowering text comments that help them feel close to others, cared for, and supported, thereby fostering cognitive improvements. Hence, we proposed the following hypothesis:

Hypothesis 1 (H1): Intimacy has a positive impact on cognitive change in support seekers in online mental health communities.

#### Emotional Polarity

The study of sentimental ideas, emotions, and emotions expressed in the literature is known as sentiment analysis [41]. An increasingly popular subject in emotion analysis, particularly in the context of mental health, is electrical polarity [42]. Pérez-Rosas et al [43] categorized the emotions in support text comments into 5 categories and discovered that high-quality counselors are more likely to convey positive feelings than low-quality counselors in high-quality counseling. Successful treatment outcomes are linked to higher levels of positive emotions [44]. As a result, showing more positive feelings in a supporting text comment may improve support seekers’ feelings and alter their cognitive processes. Accordingly, we proposed the following hypothesis:

H2: Positive emotional polarity has a positive impact on cognitive change in support seekers in online mental health communities.

#### First-Person Words

The use of personal pronouns is correlated with mental health; support seekers’ psychological conditions improve when they use the first-person singular pronouns less frequently [45]. According to cognitive behavioral therapy, support seekers undergo a cognitive change when their focus turns from themselves to others, that is, when they become more encouraging and compassionate toward others [46]. In other words, symptom reduction is correlated with a psycholinguistic shift during therapy from talking about oneself and the present to talking about other people and moments in time [47]. Sharma et al [32] explored the impact of self-focused pronouns (first person) on the counseling effect and discovered that as counseling progresses, the frequency of first-person pronoun use by psychotherapists increases. Online community members—who tend to be amateurs—often use the first or the third person to help others when they share their own comparable experiences or those of well-known individuals. This language encourages support seekers to empathize with one another and shift their viewpoint from their own difficulties to those of others by making the experiences of others feel familiar, as though someone else is experiencing challenges that are similar to their own. Therefore, we posited the following hypothesis:

H3: First-person pronoun use has a positive impact on cognitive change in support seekers in online mental health communities.

#### Future-Tense Words

Research has shown that similar to the factor of self, described in the previous section, when counselors talk about the future and help support seekers shift their viewpoint toward the future, it increases the likelihood that the support seekers will experience an altered perspective [16]. Support seekers have a better chance of surviving a crisis if they focus on the future rather than on issues from the past. We further proposed that increased use of future-oriented terms may influence support seekers to alter their viewpoint toward the future, and thus encourage cognitive change, in the setting of online mental health communities. Thus, we proposed the following hypothesis:

H4: Future temporal features have a positive impact on cognitive change in support seekers in online mental health communities.

#### Specificity

The degree to which a reference in a text can be clearly understood is referred to as its specificity. Supportive text comments with good ratings use fewer terms with an abstract connection [25]. The degree of abstraction and concreteness of online comments affects how valuable they are seen to be, which in turn influences how online users make decisions [48]. Construal level theory [49] states that people will use low-level interpretations when they perceive a target as being close or attainable but high-level interpretations when they perceive a target as being psychologically far. Low-level explanations are concrete, but high-level explanations are abstract [50]. Low-level language helps support seekers feel empathy, reduce the psychological and social distance between themselves and support providers, and become aware of the efforts being made on the part of support providers to assist them, all of which can lead to positive cognitive changes. Thus, we proposed the following hypothesis:

H5: Specificity characteristics have a positive impact on cognitive change in support seekers in online mental health communities.

#### Language Style

Many nonverbal cues, such as facial expressions and body gestures, disappear in the online interactive environment, and more written language is needed to seek or provide support compared to offline, face-to-face psychological counseling. Therefore, the acceptance of psychological support may be significantly influenced by the language style of the comments offered as support [51]. The language used during counseling affects the results in terms of the patient’s mental health. Online, a member’s level of identification with the group and engagement should be reflected in how closely their linguistic style resembles that of the entire community [52]. The function words in text comments more accurately reflect the language style than the substance of the comment [53]. Despite being few in number, function words make up 60% of the vocabulary that people use [54]. Function words can act as markers of emotional states, social identities, and cognitive styles [55]. The listener’s social and cognitive skills must be further developed due to the increased usage of function words [56]. The rising use of function words by those providing support in online mental health communities creates a need for more effort by support seekers and might not be helpful for their development. Accordingly, we proposed the following hypothesis:

H6: Language style characteristics have a positive impact on cognitive change in support seekers in online mental health communities.

Therefore, drawing on signaling theory, in this study, we proposed a framework to examine which factors affect cognitive change in online mental health communities and measured their characteristics using natural language processing (NLP) techniques. This study has potential for supporters in online mental health communities. It offers suggestions for designing and managing these communities and the interactions of their members.

Our main research contributions are as follows: First, we proposed a model to reveal factors affecting cognitive change in online mental health communities and provided empirical support. Our model was based on theories and strategies of existing counseling scenarios. Second, we extended signaling theory to psychological support scenarios in online mental health communities, proposed unique text features of psychological support from information signals and emotional signals, and verified the hypothesis. Finally, we highlighted the significance and relevance of 2 new factors, intimacy and specificity. To the best of our knowledge, these 2 variables have not been studied in online mental support scenarios. The results regarding the influence of intimacy and specificity on cognitive change conflict with previous findings and are specific in the context of psychological support in online mental health communities.

## Methods

### Data Collection

**Figure 2:**
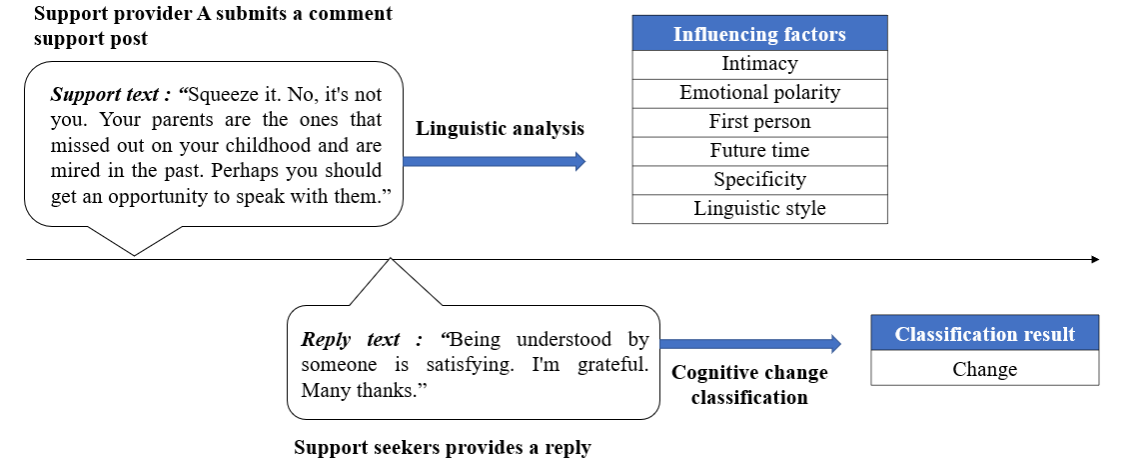
A typical sample of (support text, reply text) pairings.

We collected data from a representative online mental health community Zhihu, which is a publicly available platform where posts, comments, and replies can be seen by everyone. In May 2023, the Zhihu platform’s question-and-answer (Q&A) subcommunity “mental health” had 5.31 million followers. The Q&A text on Zhihu related to mental health was collected using a crawler, which also collected posting information, comment support information, and reply text. In the Q&A text, we primarily analyzed comment data and reply text. We collected a rich data set of 276,528 posts and 565,813 reply comments on Zhihu’s Q&A “mental health” subcommunity as of May 2022. We obtained 12,868 (support text, reply text) pairings. Figure 2 shows a typical sample, and more samples can be found in Multimedia Appendix 1.

### Dependent Variable

Gu et al [57] used the TextCNN and the Word2vec algorithm for cognitive change classification and found the best model with a word vector length of 300 and a window size of 3. Therefore, we marked the 12,868 text items as either having or not having cognitive change using the trained cognitive change classifier that was combined with emotional information. The text items that indicated a person had undergone cognitive change received a score of 1, while the text items that indicated no change received a score of 0.

The 12,868 reply comments’ sentences were categorized using a cognitive change classifier yielding 5439 pieces of data with cognitive change and 7681 pieces of data without cognitive change. Word frequency analyses of the comment text revealed the presence of words such as “you,” “me,” “myself,” and “is” in both categories of comments, suggesting that the majority of support providers provide support to support seekers by sharing their own experiences or making recommendations. More frequently, in posts by children, references to the children’s parents, friends, and family, as well as certain negative emotional phrases such as “depression” and “bad” appeared. However, words such as “come on,” “hug,” “hope,” and similar expressions appear more frequently in comments that do alter cognition [58,59].

### Independent Variables

#### Intimacy

Gilbert and Karahalios [60] compared intimacy-related terms in the LIWC dictionary to the words in text passages and counted the number of times they appeared to determine the level of intimacy in the passages. Intimacy was determined by Pei and Jurgens [61] by training NLP models through extensive text analysis to determine the intimacy of text comments. Pei and Jurgens [61] argued that text that makes people feel intimate, profound, and humanized has a high level of intimacy. The intimacy of interactive text in online health communities was predicted by Reuel et al [62] using the LIWC dictionary, the latent Dirichlet allocation (LDA) topic distribution, emotion categories, and other factors. To determine the intimacy of support stickers in our study, the Chinese LIWC dictionary’s words in the categories of family, friends, home, sexuality, swearing, work, leisure, money, body, and health were employed. Higher intimacy is indicated if the support giver is willing to discuss private matters, such as family and friends. As a result, the intimacy score is greater when the support text has more terms that indicate intimacy.

#### Positive Emotions

A Python package called SnowNLP is used to process the Chinese natural language. It supports Chinese natural language operations such as text classification, sentiment analysis, part-of-speech tagging, and Chinese word segmentation. In this work, emotion in support comments was determined using the SnowNLP package. We started by using the “pip” command to install the SnowNLP package in a Python environment. The support comment text was then used as a parameter by the SnowNLP algorithm to determine emotional polarity values. Results from SnowNLP vary from 0 to 1, with values closer to 1 denoting pleasant, positive emotions and values closer to 0 denoting negative emotions.

#### First-Person Words

The point of view of posts in an online mental health community was calculated by Lyons et al [63] using the personal pronouns in the LIWC dictionary, such as “me,” “you,” and “them.” In the context of online mental health assistance, we investigated how the language viewpoint of the support provider affects cognitive change in the support seeker. First, preprocessing of the 12,868 comments permitted text segmentation, and part-of-speech tagging was used to annotate the word segmentation results. Using personal pronouns from the dictionary, terms relating to the category of personal pronouns were screened.

#### Future-Tense Words

To discuss the impact of using a temporal perspective on cognitive change in support seekers in an online mental health community, we divided time into the past, present, and future. A dictionary was created primarily through part-of-speech tagging by extracting words designated as time related. The dictionary was then split into a past-tense word dictionary, a present-tense word dictionary, and a future-tense word dictionary through further manual screening. Finally, the dictionary statistics comments confirmed the text’s distribution of different time words. First, preprocessing of the 12,868 text items permitted text segmentation, and part-of-speech tagging was used to annotate the word segmentation results. The dictionaries created, as previously described, were then used to count the occurrence of the 3 categories of time-related words in the text items. Table 1 lists several time-related terms.

**Table 1:** Excerpts from the dictionary of words related to time.

Category	Words/phrases
Past-tense words	Years ago, the day before yesterday, in the past, ten years ago, childhood, at that time, then, that year, in the early days, when I was a child, before
Present-tense words	The present, the moment
Future-tense words	In the future, after, a little while, tomorrow, next year, after a long time, later, within two years, at the end of the year

#### Specificity

Polander and Shalin [64] discovered that online users of mental health services use psychological language in their narratives to express particular concepts, that they assess the content of particular psychology-related posts using the categories of leisure and work from the LIWC dictionary, and that they create a narrative dictionary based on the LIWC dictionary that includes causal words, time words, emotion words, and transition words. A 62,000-word-long English specificity dictionary was created by Muraki et al [65]. Similar to Muraki et al’s [65] approach, Xie and Bi [66] developed word-level and sentence-level specificity inference models based on large-scale Chinese specificity dictionaries. In our study, based on a Chinese specificity dictionary, we calculated the influence of the level of specificity of the comment text on cognitive change in support seekers. The specificity dictionary developed by Xie and Bi [66] is presented in tabular form in their study. The dictionary was transformed into a usable data format for this investigation. Key value matching was used to determine the specificity of each word in the comment text, and the average specificity of the words was derived as a gauge of sentence specificity.

#### Language Style

Function words in text can more accurately reflect the text’s language style than its substance [53]. The use of function words in online mental health communities was examined as a method of language style calculation [66-68]. Pronouns, prepositions, adverbs, conjunctions, auxiliary verbs, etc, are examples of function words that can express significant facets of the speaker’s motivation, requirements, and personality [69]. To conduct part-of-speech tagging, count words, and calculate function words as a measure of language style, the Baidu part-of-speech tagging tool LAC [70] was used in this study. This tool allows researchers to examine the impact of support providers’ language on changes in the cognition of support seekers in an online mental health community. Only the use of prepositions, adverbs, conjunctions, and auxiliary verbs was examined in this section because personal pronouns have already been covered in depth in the section on first-person words.

### Control Variables

Some support providers use the second-person point of view in their comments, to give direct instruction and counsel based on the support seekers’ experiences. The likelihood of a support seeker showing improvement, however, decreases if past-tense verbs are used excessively. Thus, cognitive change may be influenced by the use of both second-person and past-tense verbs.

Yu et al [71] defined complexity as the cognitive load of knowing a certain subject, and Nadeem and Ostendorf [72] defined linguistic complexity as the difficulty faced by language users in understanding sentences. Therapists tend to use more difficult-to-read, grammatically complicated language when counseling clients who are more worried. This language use might be connected to the need for therapists to engage in deeper communication with such clients. One study calculated the complexity of the consulting language using 12 factors, such as lexical diversity, grammatical simplicity, readability, and typicality [73]. Online support seekers in online mental health communities typically describe their experiences in detail in their postings, so support providers typically do not need to engage in more in-depth communication by using language that is more challenging to read. This situation contrasts with that of face-to-face counseling.

The mental health difficulties posted online by support seekers may change over time due to other, unidentified variables because the connections formed in the online community are asynchronous. Hence, our study used the interval between the time of the original post and the time of the supporting comments as a control variable. Additionally, those who seek support from a larger group of people may interact more frequently and feel certain psychological comforts, which influences their own cognitive improvement. As a result, this study also used the support seeker’s number of followers, the interval between the original posting and the support seeker responding to comments, and the number of supporting comments as control variables.

In summary, this study’s control variables were the use of the second person, the use of the past tense, topic complexity, the number of likes on support comments, posting and comment support times, and the number of comments. These variables were measured as follows: We calculate the number of second-person words and past-tense words by creating dictionaries. To determine the topic complexity, Lu et al [74] calculated the complexity of sentences and words. A lexical complexity prediction approach based on a 5-component Likert scale and manual annotation was proposed by Shardlow et al [75]; this method splits lexical complexity into 5 levels. By assessing the topic complexity of the text, Shin et al [76] used the LDA model (Genism package) to determine the complexity of the analysis text. Understanding certain words, certain sentences, and the variety of topics in the supportive comment text, when combined with psychological support situations, might influence cognitive change in support seekers. The number of words is used to gauge sentence difficulty after the preprocessing and word segmentation of the comment text. The number of subjects in the text is determined to gauge the topic complexity, and the LDA text-clustering technique is used to cluster the text.

Based on signaling theory, we constructed a mining model of the 6 independent variables: intimacy, emotional polarity, the use of first-person words, the use of future-tense words, specificity, and linguistic style. Cognitive change was used as the dependent variable. Table 2 displays the precise procedures used to calculate each variable.

**Table 2:** Description of variables.

Variables		Description
**Dependent variable**		
	Cognitive change	Support seekers indicate in their responses to comments whether they are getting better.
**Independent variables**		
	Intimacy	The number of intimate words in a support comment.
	Emotional polarity	The emotion degree score (in the range of 0-1, where 1 is the strongest positive emotion) in a support comment.
	First person	The number of first-person words in a support comment.
	Future time	The number of future-tense words in a support comment.
	Specificity	The degree to which a reference in a support comment can be perceived directly; the average specificity of support stickers based on the specificity dictionary.
	Linguistic style	The number of function words (adverbs, prepositions, conjunctions, and auxiliary verbs) in a support comment.
**Control variables**		
	Second person	The number of second-person words in a support comment.
	Past tense	The number of past-tense verbs in a support comment.
	Complexity	The number of words and the number of topics in a support comment.
	Interval time	The calculated time between the original post and the support comment.
	Approval number	The number of likes for a support comment.

### Statistical Analysis

We used the statistical analysis program IBM SPSS 20.0 to analyze the correlation of variables. After the multicollinearity test, we used binomial logistic regression to test our hypotheses, considering that the independent and control variables were continuous and the dependent variable was dichotomous. The robustness test was carried out by using other models, as well as a sample subset. Specifically, we used the probit model to test the effect of the independent variables on the dependent variable. We then randomly selected 50% of the sample and ran binomial logistic regression and probit model tests on a subset of the sample.

### Ethical Considerations

This study was approved by the Biomedical Ethics Committee of Hefei University of Technology (approval number HFUT20240520001H). Since the postings and comments of members of online mental health communities are related to user privacy, deidentified methods were adopted in this study: (1) After matching comment postings with reply postings, only the text of the comments and reply postings was retained, and (2) data, such as links and email addresses showing personal information contained in the text, were removed.

## Results

### Empirical Results

Multimedia Appendix 2 displays the correlation analysis results. The outcomes of the multicollinearity test in this study were tolerance and the variance inflation factor (VIF, which is the reciprocal of tolerance). After removing word complexity, no multicollinearity issue existed among the explanatory variables, because the VIF values of all of the explanatory variables were no more than 10, and thus, all explanatory variables could be used in the regression model.

To further understand the links between variables, we used a linear regression model to assess the elements influencing cognitive change. The 6 independent variables and the control variables were all continuous variables, but the dependent variable, cognitive change, was a dichotomous variable. Therefore, we used a binomial logistic regression model to investigate the elements. The findings demonstrated that at least 1 of the included variables was statistically significant. Table 3 shows the results of the regression analysis. Table 4 displays the findings of the hypothesis testing.

**Table 3:** Results of regression analysis.

Variable	β	Significance	*P* value	Exp(B)
Intimacy	–1.706	0.213	<.001	0.182
Emotional polarity	.890	0.058	<.001	2.434
First person	.120	0.014	<.001	1.127
Future time	.301	0.081	<.001	1.351
Specificity	–.018	0.005	<.001	0.982
Linguistic style	–.838	0.116	<.001	0.433
Second person	.013	0.016	.042	1.013
Past tense	–.068	0.071	.039	1.070
Complexity	–.002	0.003	.415	0.998
Approval number	.1×10^–5^	0.000	.007	1.000
Interval time	.9×10^–5^	0.000	<.001	1.000
B	.542	0.335	N/A^a^	N/A

^a^N/A: not applicable.

H1 concerned the impact of intimacy on cognitive change among support seekers in online mental health communities. The coefficient of prediction for cognitive change in the regression analysis, however, indicated that intimacy had a significant negative effect on cognitive change (β_intimacy_=–1.706, *P*<.001). Support texts with fewer privately similar experiences are more likely to result in a change for the better for the support seeker.

The impact of emotional polarity on support seekers was tested in H2. This hypothesis—that a significant emotional polarity score favorably influences cognitive improvement (β_emotional_polarity_=.890, *P*<.001) and that support seekers feel comfortable and supported when support providers display more positive emotions—was validated by the regression analysis, which showed that emotional polarity has a positive regression coefficient. Straightforward helpful messages of approbation and encouragement can cause a change in cognition and pleasant emotions in support seekers.

H3 explored the effect of using first-person terms (ie, providing a self-identity of the supporter) on cognitive change in the supporter-seeker. The hypothesis was supported by the regression results, which demonstrated that the use of the first person has a significant positive impact on cognitive change (β_first-person_=.120, *P*<.001). When support seekers are encouraged to improve by support providers sharing their own experiences or directly making suggestions, they are positively affected, leading to cognitive change.

The use of words related to the future is a temporal aspect, which was examined in H4. The results showed that the use of present- and future-tense words in support postings has a significant positive impact on the occurrence of change for the better in support seekers (β_future_words_=.301, *P*<.001). Increased use of present- and future-tense words leads to positive cognitive change in support seekers.

H5 concerns how the degree of specificity in support posts affects whether support seekers experience a change for the better. The regression resulted in a significant negative effect of expression specificity on cognitive change (β_specificity_=–.018, *P*<.001). This result implies that support comment postings containing more specific words do not cause support seekers to undergo a change for the better.

H6 examined how language style traits affect the cognitive shifts that support seekers’ experience. According to the regression analysis, the use of function words has a detrimental impact on cognitive change (β_language_style_=–.838, *P*<.001). The findings support the idea that increasing the use of function words may not improve support seekers’ ability to assist others in online health forums.

**Table 4:** Summary of hypothesis testing.

Hypothesis	Supported?
H1: Intimacy significantly positively affects cognitive change in support seekers in online mental health communities.	No
H2: Emotional polarity scores significantly positively influence cognitive change in support seekers in online mental health communities.	Yes
H3: First-person word use significantly positively affects cognitive change in support seekers in online mental health communities.	Yes
H4: Future temporal features significantly positively affect cognitive change in support seekers in online mental health communities.	Yes
H5: Specificity characteristics significantly positively affect cognitive change in support seekers in online mental health communities.	No
H6: Language style characteristics significantly negatively affect cognitive change in support seekers in online mental health communities.	Yes

### Robustness Check

We also used the probit model to examine the effects of independent variables on the dependent variable in order to evaluate the robustness of the model. Tables 5-7 contain the test results. The positive, negative, and significant impacts of the independent variables on the dependent variable were all supported by the binomial regression model. After selecting 6434 (50%) of the 12,868 data items at random, the sample subset was subjected to binomial logistic regression and a probit model test. The test results showed that both positive and negative effects of the independent variables on the dependent variable were significant and matched the effects of the total sample.

**Table 5:** Results of probit regression analysis on the full sample (N=12,868).

Variable	β	Significance	*P* value
Intimacy	–.960	0.179	<.001
Emotional polarity	.465	0.037	<.001
First person	.054	0.007	<.001
Future time	.156	0.028	<.001
Specificity	–.013	0.003	<.001
Linguistic style	–.534	0.113	<.001
Second person	.013	0.010	.196
Past tense	.028	0.042	.507
Complexity	.0023	0.002	.349
Approval number	1×10^–6^	0.000	.009
Interval time	8×10^–6^	0.000	.001
B	.018	0.043	N/A^a^

^a^N/A: not applicable.

**Table 6:** Binomial logistic regression analysis results of 50% (n=6434) of the samples.

Variable	β	Significance	*P* value	Exp(B)
Intimacy	–1.895	0.407	<.001	0.150
Emotional polarity	.759	0.087	<.001	2.137
First person	.090	0.018	<.001	1.094
Future time	.230	0.067	<.001	1.259
Specificity	–.020	0.006	<.001	0.980
Linguistic style	–1.115	0.258	<.001	0.328
Second person	.012	0.023	.599	1.012
Past tense	.139	0.099	.161	1.149
Complexity	.002	0.004	.641	1.002
Approval number	1×10^–6^	0.000	.192	1.000
Interval time	8×10^–6^	0.000	.037	1.000
B	.101	0.099	N/A^a^	1.106

^a^N/A: not applicable.

**Table 7:** Results of probit regression analysis on the full sample (N=12,868).

Variable	β	Significance	*P* value
Intimacy	–1.151	0.252	<.001
Emotional polarity	.438	0.051	<.001
First person	.048	0.010	<.001
Future time	.139	0.040	<.001
Specificity	–.011	0.004	.003
Linguistic style	–.687	0.160	<.001
Second person	.009	0.014	.491
Past tense	.080	0.059	.171
Complexity	.001	0.002	.624
Approval number	1×10^–6^	0.000	.174
Interval time	8×10^–6^	0.000	.039
B	.087	0.061	N/A^a^

^a^N/A: not applicable.

## Discussion

### Principal Findings

This study provides valuable insights into the linguistic factors involved in support seekers in online mental health communities feeling better. We studied the impacts of intimacy, emotional polarity, the use of first-person words, the use of future-tense words, specificity, and language style on cognitive change in support seekers in online mental health communities. We found that support comments with lower intimacy, higher positive emotional polarity, lower specificity, more first-person words, more future- and present-tense words, and fewer function words are more likely to make support seekers feel better. Our results show that the effects of most factors are consistent with the hypotheses, except for intimacy and specificity.

According to signaling theory [28], affective signals make support seekers feel better by increasing feelings of empathy and similarity between support seekers and support providers. Our findings show that positive emotions, the use of first-person words, and the use of future-tense words can make support seekers feel better. This result indicates that support providers can make support seekers perceive similarity and develop emotional empathy through positive emotional guidance, their own similar experiences, and directing the gaze to the future so that improvement occurs. According to information signaling [28], language style makes support seekers feel better by reducing the cost of receiving and understanding support signals. This means that support providers can reduce the use of function words.

The findings also suggest that intimacy has a negative effect on cognitive change and that support texts with less intimacy are more likely to make support seekers feel better. However, this result is contrary to existing research[37,39], which suggests that the expression of intimacy positively contributes to mental health. This result may be explained by the fact that other people in the community, such as support seekers, provide social support by expressing similar experiences [77]. These experiences stimulate the support seekers’ recollection of their own negative experiences, such as similar family environments, similar treatment experiences, or similar attitudes [78], thus preventing positive cognitive changes from occurring. However, if they encourage support seekers to elaborate on their own experiences and encourage them by expressing positive emotions, an improvement is seen, as discussed in H2.

The possible reasons the effect of specificity on cognitive change is negative are similar. Posts with more specificity often mean that support providers disclose more details of similar negative experiences. Specific words are more likely to make it easier for support seekers to recall their own similar negative experiences in the imaginal system, which is not conducive to making them feel better.

On this basis, we conclude that support providers providing social support by disclosing details of similar negative experiences is not conducive to supporting support seekers to feel better, and more positive emotions are needed to guide them toward the present and future. The result is consistent with psychotherapists’ psychotherapeutic strategy in offline counseling scenarios [32].

### Theoretical and Practical Implications

This study has 2 theoretical implications. First, we transferred signaling theory to the study of factors influencing cognitive change in online mental health communities and divided the factors into affective signals and information signals. We also verified the proposed model. The findings suggest that intimacy, emotional polarity, discussion of self, use of time-related words, specificity, and language style (use of function words) all significantly influence cognitive change in support seekers in online mental health communities. Thus, this study extended signaling theory. Second, based on previous research, but also going beyond that, this study investigated how new factors, such as intimacy and specificity, affect cognitive change. It calculated the intimacy and specificity of supporting comments by creating intimacy and specificity dictionaries, and then it confirmed the impact of these 2 factors on cognitive change using regression analysis. The outcomes of this study related to intimacy and specificity disagree with other findings; we discovered that both intimacy and specificity are associated with the psychological support provided in online mental health communities and that both are adversely correlated with cognitive change.

The research also has practical implications. Our findings encourage tailored strategies for mental health support and intervention. Online mental health community managers can use context-specific support techniques, offer real-time ideas when posting support comments, and provide information for the design of the online interface by researching variables that influence the cognitive shifts in support seekers. In addition, community support providers can customize their replies by using the best support comment tactics for each support seeker based on their unique traits. To foster relationships between support seekers and support providers, emphasis should be placed on encouraging other members of the online mental health community to write and offer assistance. Interventions can be created to allow service providers to cater their assistance to each support seeker individually. Most significantly, support seekers who have posted an issue and who are not receiving adequate support can be helped by the referral, identification, and mentoring procedures of the online health community.

### Limitations and Future Recommendations

Our study has several limitations. First, we included 1 online mental health community, and the findings may differ depending on the data sources used. Other contexts should be examined in future studies. Second, we did not set up support strategies in an actual online mental health community to verify whether they would improve the quality of support. Future research can strategically aid auxiliary support providers in writing supportive comments by analyzing and designing strategies and combinations of strategies so that the comments will better aid support seekers and lead to cognitive change. In addition, the characteristics of support seekers should be fully considered in functional design in order to further provide personalized support strategies. For example, the features of support comments that are more agreeable to support seekers with high participation levels versus those with low participation levels should be considered, as well as variations in the features of support comments used to treat various mental health issues.

### Conclusion

This study investigated elements that influence cognitive change in online mental health communities. It used psychological counselors’ face-to-face consultation techniques, described in existing research, to propose hypotheses, and it specifically applied signaling theory to identify factors that influence cognitive change. We found that support comments are more likely to alter support seekers’ cognitive processes if they have lower intimacy, higher positive emotional polarity, lower specificity, more first-person words, more future- and present-tense words, and fewer function words. The result is consistent with psychotherapists’ psychotherapeutic strategy in offline counseling scenarios.

## Appendix

Multimedia Appendix 1 Examples of support text and reply text.

Multimedia Appendix 2 Relevant analysis results.

## Abbreviations

LDA: latent Dirichlet allocation
LIWC: Linguistic Inquiry and Word Count
NLP: natural language processing
Q&A: question-and-answer
VIF: variance inflation factor

## Data Availability

The data sets generated and analyzed during this study are available from the corresponding author upon reasonable request.
