# Supplementary material for: The Impact of Linguistic Signals on Cognitive Change in Support Seekers in Online Mental Health Communities: Text Analysis and Empirical Study
Source: J Med Internet Res. 2025 Jan 14;27:e60292. doi: 10.2196/60292 (PMC11775492; doi:10.2196/60292)
Supplement: Multimedia Appendix 1 [file jmir_v27i1e60292_app1.docx]

**Multimedia Appendix 1.** **Examples of support text and reply text**

| Support text | Reply text |
| --- | --- |
| Really nice and heartbreaking! Hope you've improved! You must persevere! You are capable of doing it. Really adore you so much! May love be all around you! Always joyful! | I'll do my best. Thank you |
| You deserve a hug. It's just a phase, you know. It will pass. If you're sad, find a healthy outlet for it; don't keep it inside all the time. Too cautious and you worry about upsetting other people, but too indulgent and you will receive gentle treatment. | Thank you. I'll try and get out |
| Since I used to stammer when I was younger and have some regrets about how I looked, I can relate to your feelings of exclusion and concern for other people's perceptions. Later, I read a lot of books, which caused my mind to become overly broad. Gradually, though, it started to become better. I'm hoping we can collaborate and recharge together! | Since I struggled with anxiety, I discovered that reading is a really fantastic thing that really helps me, and that there are plenty of others working together who have had similar experiences. Added gas! |
| I have the same infection as you, with more symptoms than you but no fever. I share many of your symptoms, including enlarged lymph nodes and dread. | Don't be afraid. It'll get better. |
| Squeeze it. No, it's not you. Your parents are the ones that missed out on your childhood and are mired in the past. Perhaps you should get an opportunity to speak with them. | Being understood by someone is satisfying. I'm grateful. Many thanks |
| You are good enough for the moment❤ | The same to you[love] |
| On the good days, enjoy the moment😄 | The most crucial thing is to be joyful and live in the now. Together, cheer(⌒▽⌒) |
| You seem to see everything through the eyes of your classmates, so the story is either yours or made up | Funny. Do you not have friends? Isn't it common to discuss a breakup with someone? I spoke in my own language after she told me and gave me the conversation logs, and you claimed I made it up. Make yourself happier. |
| Your family is so tired of living, and the $300 single is pushed around | You only saw $300 |
| Wanting to fit in is difficult; someone who lacks a strong heart is not a whole person. | Nobody is able to experience another person's feelings. Just reflect on those ideas and words. It doesn't need to be stated. |
| Not hating yourself. You are just a pure lazy man | Are you judging me? |
