# Supplementary material for: The Impact of Linguistic Signals on Cognitive Change in Support Seekers in Online Mental Health Communities: Text Analysis and Empirical Study
Source: J Med Internet Res. 2025 Jan 14;27:e60292. doi: 10.2196/60292 (PMC11775492; doi:10.2196/60292)
Supplement: Multimedia Appendix 2 [file jmir_v27i1e60292_app2.docx]

**Multimedia Appendix 2. Relevant analysis results**

|  | cogchange | Intimacy | Emotional polarity | First person | Future time | Specificity | Linguistic Style | Second person | Past tense | Complexity | Interval time | Approval number |
| --- | --- | --- | --- | --- | --- | --- | --- | --- | --- | --- | --- | --- |
| cogchange | 1 |  |  |  |  |  |  |  |  |  |  |  |
| Intimacy | –0.040^b^ | 1 |  |  |  |  |  |  |  |  |  |  |
| Emotional polarity | 0.138^b^ | 0.088^b^ | 1 |  |  |  |  |  |  |  |  |  |
| First person | 0.103^b^ | 0.053^b^ | 0.103^b^ | 1 |  |  |  |  |  |  |  |  |
| Future time | 0.100^b^ | 0.002 | 0.123^b^ | 0.446^b^ | 1 |  |  |  |  |  |  |  |
| Specificity | 0.047^b^ | 0.098^b^ | 0.077^b^ | 0.696^b^ | 0.430^b^ | 1 |  |  |  |  |  |  |
| Linguistic Style | –0.036^b^ | –0.049^b^ | 0.017 | 0.023^a^ | 0.017 | –0.015 | 1 |  |  |  |  |  |
| Second person | 0.046^b^ | 0 | 0.110^b^ | 0.300^b^ | 0.163^b^ | 0.297^b^ | –0.007 | 1 |  |  |  |  |
| Past tense | 0.057^b^ | 0.01 | 0.085^b^ | 0.466^b^ | 0.320^b^ | 0.448^b^ | 0.019 | 0.115^b^ | 1 |  |  |  |
| Complexity | 0.085^b^ | 0.073^b^ | 0.167^b^ | 0.686^b^ | 0.436^b^ | 0.687^b^ | 0.037^b^ | 0.335^b^ | 0.378^b^ | 1 |  |  |
| Interval time | 0.026^b^ | –0.017 | 0.005 | 0.003 | –0.007 | –0.009 | 0.015 | –0.021^a^ | –0.010 | 0.002 | 1 |  |
| Approval number | 0.037^b^ | –0.010 | 0.004 | 0.028^b^ | 0.024^a^ | 0.019 | 0.003 | 0.004 | 0.01 | 0.029^b^ | 0.014 | 1 |

^a^Significant at .05.

^b^Significant at .01.
